# Translational research in diagnosis and management of soft tissue tumours

**DOI:** 10.1186/s40644-016-0071-7

**Published:** 2016-06-07

**Authors:** Eugenio Rimondi, Maria Serena Benassi, Alberto Bazzocchi, Alba Balladelli, Giancarlo Facchini, Giuseppe Rossi, Sophie Taieb, Daniel Vanel

**Affiliations:** Diagnostic and Interventional Radiology, Istituto Ortopedico Rizzoli, Bologna, Italy; Laboratory of Experimental Oncology, Istituto Ortopedico Rizzoli, Bologna, Italy; Interventional Angiographic Radiology, Istituto Ortopedico Rizzoli, Bologna, Italy; Centre Oscar Lambret, Lille, France; Research Department, Istituto Ortopedico Rizzoli, Bologna, Italy

**Keywords:** Sarcoma, Biomarkers, Signalling pathways, Molecular imaging, Magnetic resonance imaging, Ultrasound elastosonography

## Abstract

Finding a soft tissue mass in the superficial regions is a common event in daily clinical practice. Correct management of the diagnostic process is crucial to avoid blunders. Diagnosis is posed by pathology, although both imaging and a better understanding of the cellular and molecular mechanisms play an important a role in the characterization, staging and follow-up of soft tissue masses. Cellular and molecular mechanisms can explain either the development of chemo-resistance and the underlying pre- and post-surgery metastasis formation. These are mandatory to improve prognosis and unveil novel parameters predicting therapeutic response. Imaging mainly involves ultrasound and MR and is fundamental not only in diagnosis but also in the first step of therapy: the biopsy. Novel imaging techniques like Ultrasound Elastosonography, Dynamic Contrast-Enhanced MR imaging (DCE), Diffusion Weighted MR imaging (DWI) and MR Spectroscopy (MRS) are discussed. This paper aims at reviewing and discussing pathological methods and imaging in the diagnosis of soft tissue masses underscoring that the most appropriate treatment depends on advanced molecular and radiological studies.

## Background

Soft tissue sarcomas (STS) represent 1 % of all malignant tumours [1, 2] with more than 50 histological subtypes associated with distinctive clinical and/or molecular profiles, prognosis and response to tailored therapy. STS are mostly benign with an incidence of 100 to 1 and 75 % involve limbs. Malignancy increases with age, and is significantly higher in adult patients compared to children where 75 % of masses are benign [3].

An important percentage of malignant lesions rapidly metastasize leading to a poor outcome. Despite advances in therapy, high-grade STS are associated with a 50 % mortality rate [4, 5].

Surgical removal of tumours in association with radiation and chemotherapy has brought an increase in the 5-year disease-free survival of localized STS, while clinical outcome of patients with advanced or metastatic disease remains strongly unfavorable. Metastasis are usually located in the lung. However, a significant amount of STS are associated with bone metastases and this along with adverse histological and radiological indicators are considered predictive factors for mortality [6, 7]. In order to improve the sarcoma grading and prognosis, Chibon et al. [8] established a “complexity index in sarcoma” (CINSARC) by relating gene expression to mitosis and chromosome management. Recently, multicentric studies confirmed the relationship between multidrug resistance factors and STS patient survival [9].

Based on cytogenetic and genomic data, STS are divided into “STS with simple genomics” (SSG), displaying specific genetic alterations such as chromosome translocations, and “STS with complex genomics” (SCG), with nonspecific multiple genomic alterations and a high genomic instability [10]. In the SSG group (Table 1) fusion gene products may be useful in differential diagnosis [11]. The majority of these chimeric proteins are transcription factors that cause dysregulation of target genes and this makes it difficult to apply new therapeutic tools such as antibody therapy. However, some fusion genes induce activation of tyrosine kinase end-points or autocrine growth factors that are suitable for pharmacologic inhibition [12]. The pathobiology of SCG tumours (Table 1) is still unknown, and poses challenges in diagnosis and therapeutic management.

**Table 1:** Soft tissue sarcoma molecular subtypes

STS with simple genomics	More frequent translocations	STS with complex genomics
Ewing sarcoma family (ES/PNET)	t(11;22)(q24;q12)	Malignant peripheral nerve-sheath tumor (MPNST)
Desmoplastic Small Round Cell Tumor (DSRCT)	t(11;22)(p13;q12)	Undifferentiated Pleomorphic Sarcoma (UPS)
Alveolar soft part sarcoma	t(X;17)(p11;q25)	Fibrosarcoma (FS)
Congenital fibrosarcoma	t(12;15)(p13;q25)	Pleomorphic Liposarcoma (PLPS)
Myxoid Liposarcoma (LPS)	t(12;16)(q13;p11)	Leiomyosarcoma (LMS)
Malignant melanoma of soft parts	t(12;22)(q13;q12)	Embryonal/Pleomorphic RMS (ERMA/PRMS)
Synovial sarcoma (SS)	t(X;18)(p11.23;q11)	Angiosarcoma Myxofibrosarcoma (MFS)
Alveolar Rhabdomyosarcoma (ARMS)	t(2;13)(q35;q14)	Osteosarcoma (OS)
DermatoFibroSarcoma Protuberance (DFSP)	t(17;22)(q22;q13)	
Epithelioid hemangioendothelioma	t(1;3)(p36.3;q25)	
Mesoblastic nephroma	t(12;15)(p13;q25)	
Clear cell sarcoma (CCS)	t(12;22)(q13;q12)	
Angiomatoid fibrous histiocytoma	t(12;16)(q13;p11)	
Low grade fibromyxoid sarcoma	t(7;16)(q32;p11)	
Endometrial stromal sarcoma	t(7;17)(p15;q21)	
Inflammatory myofibroblastic tumor	t(1;2)(q25;p23)	
Giant-cell fibroblastoma	t(17;22)(q22;q13)	
Extraskeletal myxoid chondrosarcoma	t(9;22)(q22;q12)	

To date, high histological grade, deeply seated and greater than 5 cm in size are universally established risk factors for STS metastatic progression. In these cases magnetic resonance (MR) imaging can help define lesions with an atypical appearance [13]. Imaging is of outstanding importance particularly in STS where novel techniques like ultrasound elastosonography, dynamic contrast-enhanced MR imaging (DCE), diffusion weighted MR imaging (DWI) and MR spectroscopy (MRS) are essential for a better understanding of the lesion.

In contrast molecular biomarkers for STS patient stratification useful as targets for tailored molecular therapies are not yet well documented.

Given these evidences, a multidisciplinary approach combining molecular aspects with pathological, radiological and clinical features is required to understand specific defects leading to metastasis formation and development of chemo-resistance in distinct STS subsets.

## Review

### Cell signalling pathways and molecular targets

Sarcomas are a heterogeneous group of mesenchymal tumours where molecular studies demonstrated biological differences even in tumours with the same diagnosis that share many histological and MR imaging features, but have a different prognosis and therapeutic strategies [11, 13].

This requires a new classification that relies on the definition of distinct biological entities followed by the need to stratify high-risk patients for whom more appropriate therapies should be planned. In the setting of malignant phenotype different cellular signalling pathways drive metastatic progression converging into common interconnection endpoints. Although consensus is emerging that treatment should be histology-driven, recent studies suggest tailored therapies against these common molecular targets [14–16] identifying the effects of genetic aberrations on downstream signalling pathways with activation of key intracellular mediators that may represent targets for biological therapies.

**Fig. 1:**
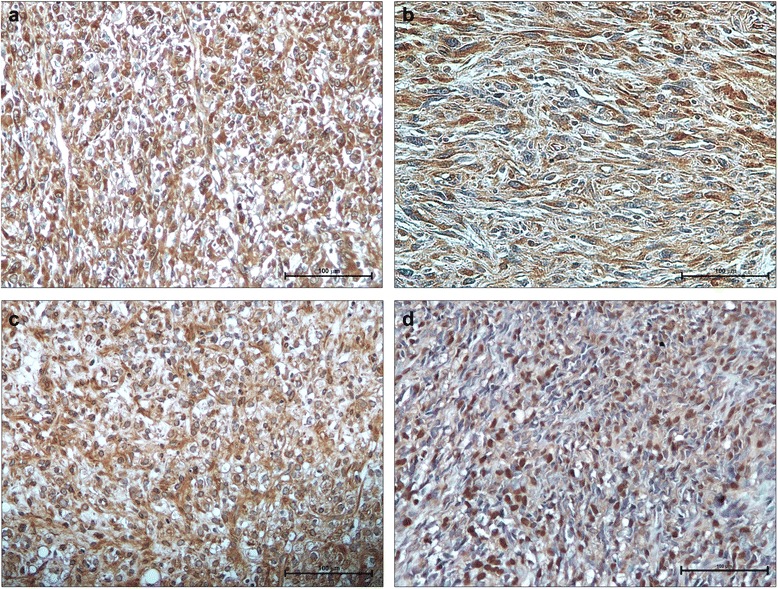
Immunohistochemistry expression of tissue biomarkers in STS. Strong and uniform immunostaining for MMP14 in leiomyosarcoma (**a**), and for VEGF in undifferentiated pleomorphic sarcoma (**b**). PDGF expression in cytoplasm of myxoid liposarcoma (**c**). Nuclear staining of IGF-1R appeared correlated to poor outcome in synovial sarcoma (**d**). (IHC, 20X)

Few highly recurrent driver genes have been described in sarcomas with high genomic complexity [17], including defects in oncosuppressor genes *RB1* and *PTEN*, mutations in *TP53* and homozygous deletions of p16/*CDKN2A,* a cyclin-dependent kinase inhibitor [18, 19]. Numerous gains and losses of chromosome DNA sequences characterize poorly differentiated sarcomas as leiomyosarcoma (LMS), pleomorphic rhabdomyosarcoma (RMS), pleomorphic liposarcoma (LPS), undifferentiated pleomorphic sarcoma (UPS) and are accompanied by rearrangements and mutations that trigger activation of downstream pathways and cell cycle perturbation [17–20]. In agreement with these data, a comprehensive analysis of a large series of sarcomas with complex genomics recognized multiple interplays between *RB1, PTEN, DKK1* signalling pathways controlling the oncogenesis process and cell proliferation [10]. Aberration in *TP53* and *p16/CDKN2A* oncosuppressor genes, growth factor signalling pathway activation and increased proteolitic and angiogenesis activity contribute to metastatic progression. Metalloproteinase activity destroys extracellular matrix promoting loss of cell-cell and cell matrix interaction, while vascular endothelial growth factor stimulates angiogenesis and trans-endothelium migration (Fig. 1a, b), playing a prognostic role in STS progression. Small tyrosine kinase inhibitors targeting vascular and fibroblast growth factor receptor are presently available for clinical use in STS subtypes including LMS, LPS and angiosarcoma. Alternative therapies targeting Hedgehog, Wnt, and Notch signalling pathways are being currently developed [21]. In accordance with the CINSARC classification that correlates gene expression related to genome complexity with metastatic progression [8], recent studies revealed differences in gene expression profile that differentiate non-translocation associated STS into prognostic subsets with a different metastatic potential [22, 23]. These data support the hypothesis that a comprehensive genetic analysis is required to stratify STS patients for therapy and clinical management [24].

Histological and morphological similarities in biologically heterogenic STS may become a challenge in posing a differential diagnosis. By using an array approach, Subramanian et al. [25] demonstrated that the expression profile of noncoding microRNA (miRNA) was unique for each type of tumour defining some biological differences useful in sarcoma classification. It is well known that mRNAs post-transcriptionally repress gene expression by recognizing complementary target sites and this makes them one of the largest families of genome regulators.

Recently, we identified differentially expressed miRNAs in a series of poorly differentiated sarcomas and recognized associated chromosome regions and gene targets that may improve differential diagnosis [26].

In STS with simple karyotype, genomic aberrations are rare and the presence of gene specific alterations as *KIT* mutation in GIST and translocations establish constant diagnostic criteria. Secondary mutations occur during metastatic progression.

The biological separation between well-differentiated LPS and myxoid LPS relies on mutually exclusive genetic alterations. Well-differentiated LPS present amplification of chromosome region 12q13-15 that address to a therapeutic strategy with anti- CDK4 and MDM2 inhibitors, while myxoid LPS is characterized by chromosomal translocation t(12;16)(q13;p11) resulting in the FUS-DDIT3 chimeric gene that plays a critical role in LPS pathogenesis.

During malignant progression from well-differentiated LPS and myxoid LPS to de-differentiated and round-cell histotypes respectively, the secondary genetic mutations lead to an increased genomic complexity, multiple numerical and structural chromosome aberrations and loss of specific targets [17]. Immunohistochemical analyses carried out on myxoid/round cell LPS specimens showed higher expression of platelet-derived growth factor receptor (Fig. 1c) in metastatic compared to localized lesions [27].

The interaction between fusion genes and signalling pathways has been fully studied in synovial sarcoma (SS) providing indication for combined therapies. The majority of patients with SYT/SSX1 had overexpression of HER2/*neu* oncoprotein associated with poor outcome [28]. In vitro studies showed high expression of insulin growth factor receptor IGF-1R and loss of function of *PTEN* in SS18-SSX -positive tumours [29, 30]. Since the central role of SS18-SSX fusion oncoprotein in tumorigenesis involves its interaction with the transcription factors ATF2 and TLE1 [31], a treatment with histone deacetylase (HDAC) inhibitors was suggested [30, 31].

After a retrospective analysis of a series SS patients [32], we correlated the expression of some potential biomarkers with clinical parameters and found that nuclear expression of IGF-1R (Fig. 1d) and chemokine receptor CXCR4 together with age, tumour size and use of radiotherapy resulted to be strongly independent adverse prognostic factors for overall survival [33]. This agrees with the observation that CXCR4 is implicated in sarcoma development and is considered a prognostic marker for poor clinical outcome [34]. Currently, immunotherapeutic strategies are promising anticancer effects in SS and in myxoid/round cell liposarcoma that present a high expression of immunogenic NY-ESO1, also useful for the differential diagnosis from other mesenchymal tumours [35].

Clinical trials using NY-ESO-1-targeted immunotherapy with genetically modified T-cells reported a clinical response in malignant melanoma and synovial sarcoma patients [36].

The link between genetic alterations and therapeutic strategies has been emphasized in other translocation-related sarcomas as Ewing’s sarcoma and alveolar RMS where the respective fusion products, EWS-FLI1 and PAX3-FOXO1, inducing activation of IGF-1R pathway stimulate proliferation in vitro and vivo [37]. Although also fusion negative RMS may present a high IGF expression, the real effectiveness of small molecules or antibodies directed at IGF-1R receptor is still under investigation [38].

In conclusion, emerging genomic and genetic approaches are being used for a predictive signature for metastases and clinical outcome [8, 14, 17].

**Fig. 2:**
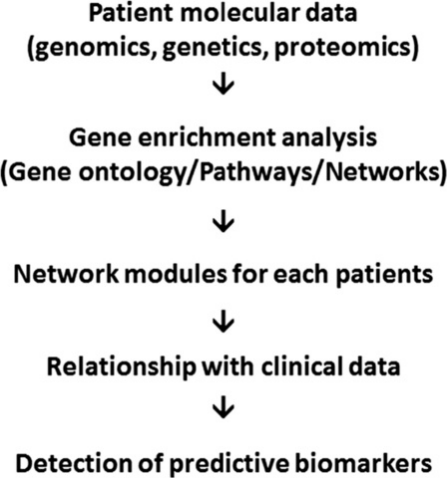
Research workflow. Data integration for clinical-molecular classification of STS

The integration with functional protein expression will create a system biology analysis able to reveal common metastatic pathways and new therapeutic target discovery [14] (Fig. 2).

### Role of the imaging

#### Ultrasound

**Fig. 3:**
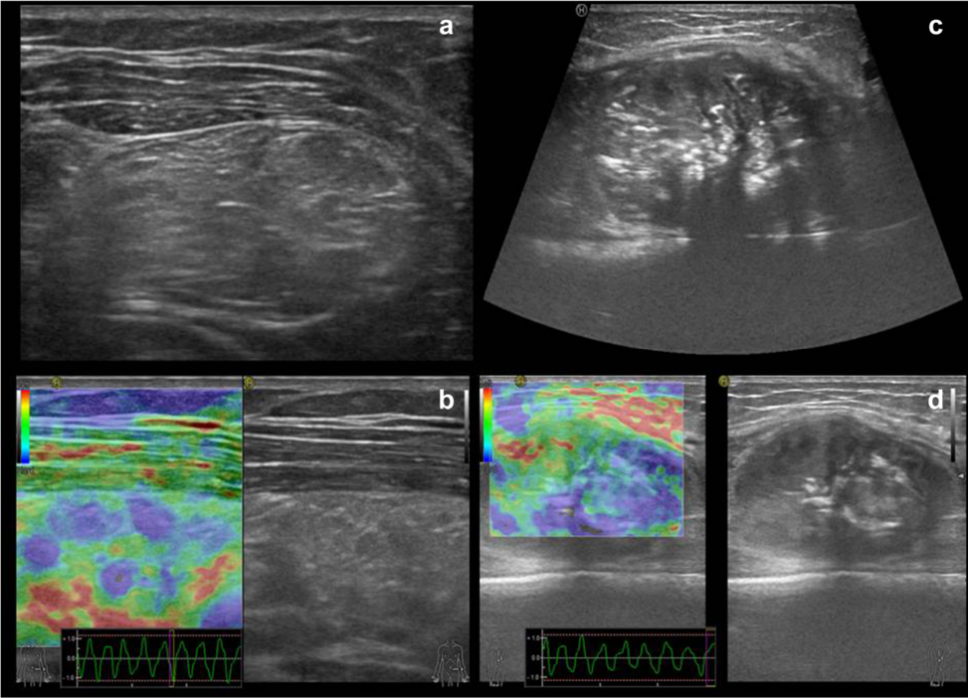
Ultrasound imaging. B-mode and elastosonographic evaluation of intramuscular lipoma (**a**, **b**) and osteosarcoma involvement of soft tissue (**c**, **d**), with calcifications included within the latter lesion. In the box of elastosonography (**b** and **d**) a colour map ranging from *blue* to *red* represents tissue elasticity; *blue* is associated with stiffness, *red* with softness

US plays an important role in detecting soft tissues masses, and with the use of Power Doppler and the recent introduction of echographic contrast media it well reveals the presence of intrinsic flow within the mass and its characteristics improving the Doppler signal. US specificity is not high, in some cases it is specific in confirming the hypothesis of a benign lesion like Baker cyst, ganglion, angioma, lypoma and ossifying myositis. In children sonography is in most cases the first imaging evaluation, and this is particularly useful for small and superficial lesions. It is a real time examination that can be carried out without problems of motion artifacts, and this is of utmost importance in children. Furthermore, dynamic scans can be helpful in the differential diagnosis (for example between STS and muscular hernias) [39]. Elastosonography is a new frontier of US, it allows to differentiate the grade of elasticity of a soft tissue mass compared to the adjacent tissues by a color map related to the compressibility of the mass. When elasticity is soft the lesion is generally benign, when less-soft it is borderline and when no elasticity is seen the lesion is malignant [40]. US elastosonography, together with B-mode, power and color Doppler can be a useful guide not only for biopsy but also in the follow-up of patients with soft tumours [41] (Fig. 3a-d).

#### Magnetic resonance imaging: traditional MR

**Fig. 4:**
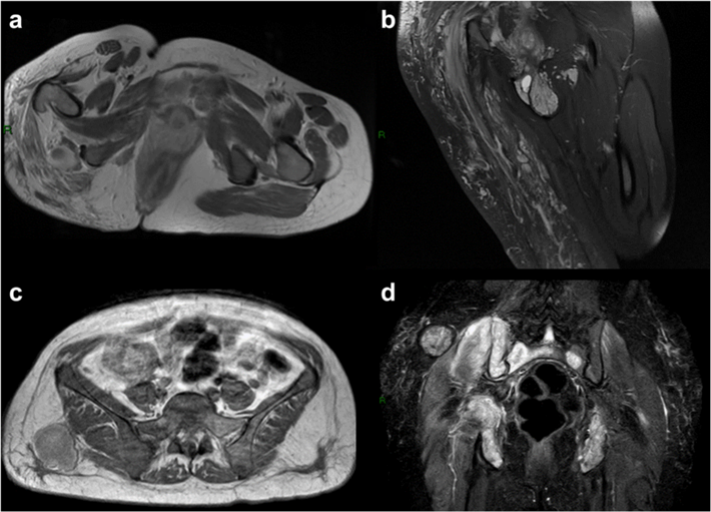
Traditional MR imaging. Axial T1 with contrast media and coronal DP fat of huge angiomatosis of the gluteal region and upper right tight. Either in T1 with contrast or in DP fat weighted image is possible to recognize the huge, mingled, and interspersed, vessels proliferation (**a**-**b**). Metastasis of angiosarcoma of the breast involving either the soft tissue of the gluteal region or the bone of sacral and iliac wing (**c**-**d**), axial T1 with contrast media and coronal STIR. In T1 only the soft tissue lesion is hyperintense, in STIR both lesion, soft tissue and bone are hyperintense

Having almost replaced CT, MRI is the method of choice for detection, characterization and follow-up of soft tissues masses. Contrast in soft tissues is of higher quality allowing an easier detection of the lesion, and improves delineation of their extent and involvement of neurovascular structures and medullary bone [41]. Many STS have a non specific behaviour in T1 and T2-weighted images. A correct diagnosis can be made only by evaluating signal intensity, intrinsic lesion features such as site, size, and growth pattern. Contrast media may help differentiate cystic and necrotic components from those of solid tumour as well as assessing lesion aggressiveness. Today in literature there is no concordance about how traditional MR imaging can precisely differentiate benign from malignant lesions [42, 43] (Fig. 4a - d).

#### Magnetic resonance imaging: new MRI techniques

As for bone tumours, newer MRI techniques such as DCE, DWI, MR Spectroscopy MRS can be used not only to cope with the problem of differentiating between benign and malignant tumours but also to improve the possibility of a correct diagnosis [44, 45]. Quantitative DCE or DCE is a non invasive technique that estimates the percentage of necrosis in malignant tumours by comparing pre- and post- treatment examination. This new technique can identify not only post treatment necrosis but also early changes during treatment to modify ineffective therapies. In STS voxels of the necrotic area enhance slowly compared to those of the remaining viable area. In DCE, although the estimate of necrosis is obtained from the whole tumour volume, results are reliable and superimposable to histopathological evaluation obtained from micron thick sections of the resected specimen [46]. DCE is also used to differentiate benign from malignant soft tissue lesions [47]. Malignant lesions usually reveal an increased rate of enhancement of vascularity compared to the benign with a DCE ratio of 3:1 or 4:1 at the first passage [45, 48]. However, DCE poses a problem with highly cellular benign tumours that overlap with STS [44].

DWI is an MR technique that is sensitive to the random motion of water. Enhancement identifies areas of inflammatory post-surgical change as well as recurrence of disease. Unlike morphological MR imaging sequences and DCE, adds functional information about tissue composition without intravenous contrast [46]. DWI techniques depict tissues with signal intensity that vary in proportion to the average distance by which water molecules are displaced per unit time through the processes of water self-diffusion. DWI is based on the principle that diffusion of the water molecules produces a net dephasing of the spinning protons within a voxel resulting in a reduced signal intensity and image brightness. When examining volume by DWI, signal intensity decreases as the average speed of water, which travels along a temporarily generated magnetic field gradient increases. The quantity of water molecules displaced is described by the diffusion constant which varies with the direction. When the distance of water displacement is the same in all directions diffusion is isotropic. DW images are direction independent, that is, they are independent from the direction along which the magnetic field is applied. In the human body this occurs in fluids where mobile water molecules are free to move in all directions like CSF, brain ventricules or fluids in cystic lesions. The diffusion constant and average water displacement in these fluids are high and equal in all directions. The result: strong signal attenuation (dark) on DWI. In structures where the movement of water molecules is restricted by body structures diffusion is not the same in all directions, but is direction dependent or anisotropic. For example, brain water molecules diffuse faster along the axon with intact myelin sheaths than perpendicular to them. In other words diffusion signal loss is higher when the gradient is applied along the axons axis and lower when applied perpendicular. This technique was first used to detect acute brain ischemia, but during the last decade it has become an imaging biomarker for oncology applied to the entire body. Today DWI is used to identify and detect tissues where pathologic processes have altered the motion of fluids outside vessels and capillaries. However, unfortunately, there is no evidence relating imaging to genetic mutations, VEGF expression, KI 67 expression, and to several other relevant markers.

DWI is acquired with an echo-planar imaging sequence. Dephasing is produced by applying addition diffusion sensitizing gradients during the image acquisition cycle. Two magnetic field gradients with the same polarity are delivered between the excitation pulse and signal collection to sensibilize the sequence to diffusion effect. In the middle of the two gradients a 180° RF pulse is delivered to change the spin phase along the field gradient direction, thus all spins involved and displaced in the diffusion process, experience a different phase shift. Protons in water molecules that have moved will be found in a different position and field strength during the second gradient. These will not be completely rephrased and their magnetic moment will no longer add thus leading to a loss of signal. It is this reduction in signal intensity that produces a difference in contrast between the moving molecules (loss of signal intensity: dark) and not moving molecules (high signal intensity: bright). Signal attenuation depends on strength and duration of the gradient pulses, their temporal separation and the diffusion constant along the direction of the gradient field. The so called b-value quantifies the amount of signal loss with a given pulse sequence and for a given diffusion constant i.e. how sensitive a sequence is to diffusion effects. The diffusion constant in biological tissues can be measured by repeated scanning with different b-values but identical parameters, in particular unchanged gradient direction. Observed diffusion constants are indicated like Apparent Diffusion Coefficents (ADCs) to differentiate them from the constant of unrestricted diffusion in pure water. Using ADCs the so called ADC maps can be built: a grey scale represents the mean ADC of the corresponding voxel. It is important to note that an area of viable tumour bright on a DWI image (for reduced water mobility both for high cellularity and membrane integrity), will be dark on the corresponding ADC map (for its lower diffusion constant). Diffusion of water is in fact more restricted in tumours than in normal tissues and this on DWI is seen as a high signal intensity in viable tumours. DWI and ADC maps provide qualitative and quantitative information about tissue cellularity and cell integrity. Potentially this is helpful in identifying not only benign from malignant lesions but also in revealing necrotic tumours and peri tumoural edemas from residual viable tumours underscoring the efficacy of tumour response to therapy. DWI is highly sensitive to motion, in brain imaging particularly to rotation or trembling of the head, in trunk imaging to respiratory motion. To cope with these drawbacks DWI uses a single shot or multi shot Echo-planar imaging (EPI). It is fast and renders this techniques less sensitive to patient motion with the advantages of covering a large volume, a high signal to noise ratio and a low power deposition in tissues because several echoes are acquired after a single excitation pulse. Multishot EPI reduces the susceptibility artifacts although it increases sensitivity to motion and scan time, while single shot fast spin-echo (RARE or HASTE) are less sensitive to susceptibility but have more limitations in signal to noise ratio and blurring. Sometimes in clinical practice differentiation of benign or malignant tumours only on ADC quantification is not so easy, while non myxoid malignant tumours show significantly lower ADC values than the benign. In myxoid tumours the differentiation is not so clear for the long T2 value of the myxoid extracellular matrix. In these, ADC values of benign and malignant tumours overlap: not all malignant tumours present more cellularity than the benign and the benign often have an extracellular matrix similar to the malignant [46–49]. This happens because conventional ADC values are calculated on a vast range of b value (b 0-600 s/mm2) and the low b values are sensitive to perfusion determined by the tumour extracellular water component as opposed to the use of high b values (300,500, 600 s/mm2) that are perfusion insensitive so perfusion effects tend to be canceled. This metric measure is called Perfusion Insensitive Diffusion Coefficent (PIDC). Costa et al. propose a PIDC value of 1.1 × 10ˉ3/s to distinguish malignant from benign lesions: malignant under this value, benign over. PIDC maps provide more accurate information about tumour tissue cellularity by minimizing vascular contributions which are higher in malignant tumours. Costa and others obtained the highest PIDC values in myxoid tumours: myxoma, myxoid liposarcoma, and low grade myxofibrosarcoma show mean PIDC values of 2.92 x 10-3 mm2/s. This value is due to the high mucin and low collagen contents of the tumour that is largely composed of water as confirmed by histological analysis [50]. When the following features are present amongst STS we can consider myxoid tumours and principally myxoid liposarcoma the main diagnostic hypothesis:

- Features of a cystic lesion on conventional no contrast MRI.
- Features of a solid lesion on DCE.
- High PIDC values with easy diffusion ADC maps on DWI.

These three features are very important because myxoid liposarcomas (about 50 % of all liposarcomas) have less than 10 % mature fat, that is a low/intermediate signal intensity on T1-weighted images on conventional MR.

Small round blue cell tumours (SRBC) are a group of less differentiated and aggressive embryonal tumours with similar histologic features and immunochemistry. They include neuroblastoma, rhabdomyosarcoma, non-Hodgkin lymphoma, Ewing sarcoma. These tumours show more restricted diffusion than other STS. For this reason the differential diagnosis of a tumour with restricted diffusion on ADC maps and very low PIDC values, small round cell tumours should be the main diagnostic hypothesis, when conventional MRI and CT both with and without contrast give clues to this hypothesis.

Fibroblastic, myofibroblastic and fibrohistiocytic tumours, the most common in all age groups, usually present low signal intensity in all sequences and a flimsy to moderate signal intensity increase after contrast on conventional MRI. These signs together with increased PIDC values and a restricted diffusion on ADC maps can help in the differential diagnosis between benign and malignant masses with morphological features of fibrous tumours on conventional MRI. It must be underlined that that there are not differences in PIDC values between benign and intermediate fibrous tumours.

It is also possible to differentiate necrotic masses such as hematomas and abscesses from necrotic hemorrhagic in malignant soft tumours by ADC map values both in the central component and on the peripheral rim. Malignant tumours for their high cellularity have a more restricted diffusion on ADC maps in the peripheral rim than in the central necrotic area. This measure associated to the typical morphological features of hematomas on conventional time based T1- and T2- MR images allow to differentiate benign from malignant necrosis.

Giant Cell Tumour is a bone and soft tissue tumour presenting low PIDC values and restricted diffusion on ADC maps. These parameters can be useful in the diagnosis and management of local recurrences by revealing differentiation of post-surgical fibrosis from recurrences [51].

**Fig. 5:**
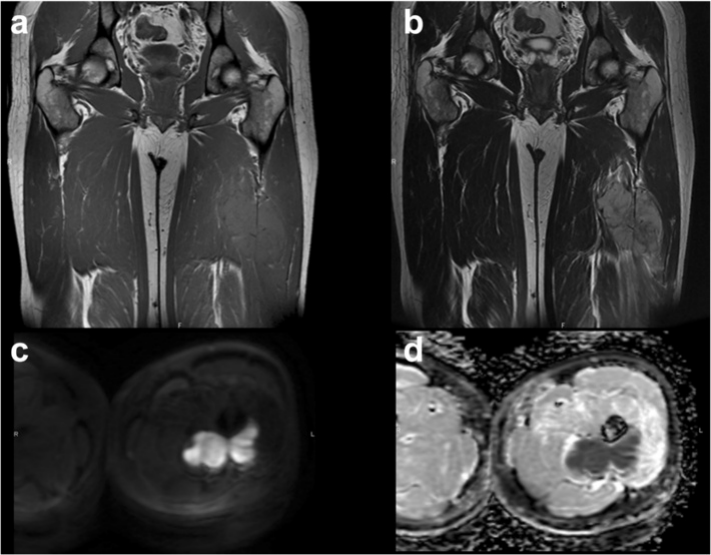
Thirty nine years old girl with a primitive neuroectodermal tumour (PNET). Coronal T1 weighted image (**a**), Coronal T2 weighted image (**b**), DWI B1000 (**c**-**d**)

**Fig. 6:**
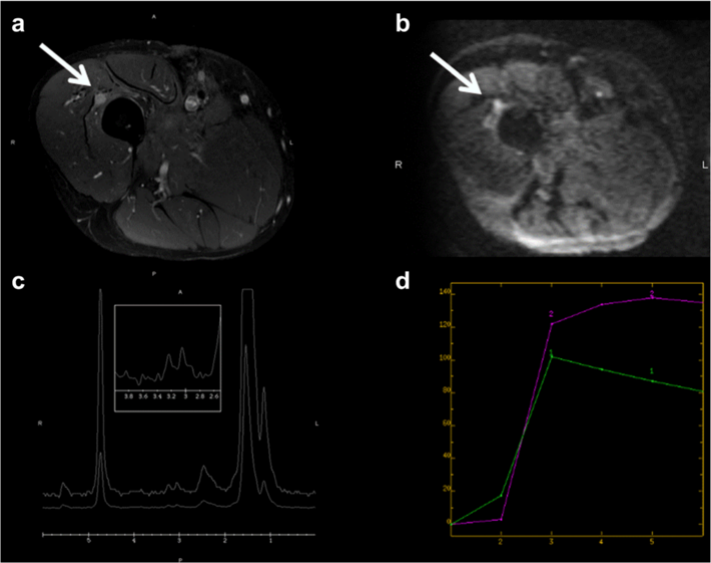
Fifty six year old man with a recurrence of an undifferentiated sarcoma grade 3. T1 fat sat weighted image with gadolinium (**a**), DWI B 1000 (**b**), spectroscopy (**c**), Perfusion contrast dynamic curve: 1 muscle, 2 lesion (**d**)

DWI together with conventional MR imaging and CT can help differentiate between pus-like fluids and serous cystic fluids. Serous and pure water like necrosis show active water diffusion and high ADC values, purulent pus-like fluids show restricted water diffusion and low ADC values for the high viscosity and cellularity of pus (Fig. 5a-d) (Fig. 6a-d).

### Imaging biomarkers

As in bone sarcomas the knowledge of tumour biology has been reinforced by advances in the field of molecular imaging that allow visualization of cell metabolic functions with the use of targets that include cell membrane receptors and enzymes of intracellular transport. The signal for imaging origins from the target molecules and their derivates [46]. A former precursor of functional metabolic imaging was bone scan with 99 metastable TC-MDP, a nuclear isomer complex introduced in the early seventies and still in use, nevertheless others are available today. However, this radiopharmaceutical agent is more sensitive for primary or secondary lesions of the bone. Procedures that follow this scheme are positron emission tomography (PET), MR with molecular MDC, optical imaging and single photon emission tomography (SPECT). Some are used only in research like optical imaging while others are used pre-clinically and clinically [52].

**Fig. 7:**
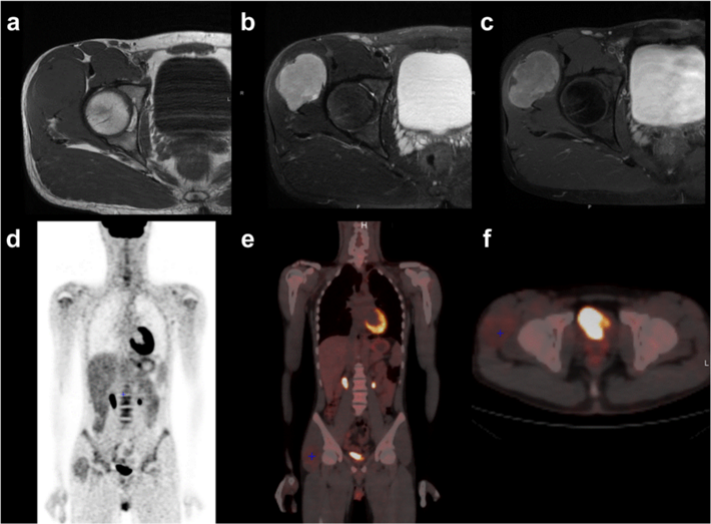
Sixteen-year-old, girl with a synovial sarcoma. T1 weighted image (**a**), T2 weighted image (**b**), T1 fat sat weighted image with gadolinium (**c**), PET Coronal scan (**d**-**e**), PET axial scan (**f**)

Fluorodeoxyglucose positron emission tomography (FDG-PET) is the method of choice in molecular imaging studies, although its role in the workup of soft tissue tumours is still to be established [53]. FDG-PET uses positron emitting radionuclides biological tracers (Carbon 11, Fluorine-18, Technetium-94 and Copper-64). FDG-PET is used as tracer to study cell metabolism tied to glucose, frequently altered in neoplasms with an increased cell turnover. FDG-PET often has a higher sensitivity than tumour markers (for example CEA) present. In cancer it is used to locate primary tumour when metastases are present and to stage tumour and its response to therapies. A decrease in FDG uptake indicates improved survival, while increased or high uptake reflects recurrences or a biologically aggressive behaviour. PET/CT fusion images help match metabolism and morphology and guide biopsy to target areas that may result in a higher diagnostic yield [46]. FDG-PET is promising in helping differentiate benign from malignant lesions, especially in bone. However, it is important to underline that also in STS there is some overlap in uptake values [54] (Fig. 7a-f).

The introduction of powerful high field MR scanners, 3 Tesla in clinical practice, up to 7–9 Tesla in clinical research, has led to two main advantages. The first is a better spatial resolution that detects smaller lesions before undetectable. The second is a stronger chemical shift disparity that makes MRS an even more unique window to cellular metabolism by non-invasively detecting the concentration of small mobile biological components. In vivo MRS allows, either with a single voxel or multi-voxels study, to identify and quantify the metabolites present within the body volume studied revealing simultaneously anatomic and physiologic information. MRS can isolate and examine the body volume without interference from the nearby structures. It can be used to measure the level of different metabolites giving quantitative chemical information reflecting the molecular composition of a tumour. Changes in metabolite level may be useful indicators of therapeutic response or of recurrence. Moreover, in molecular imaging the intrinsic contrast can be improved by the use of targeted contrast agents in both experimental and clinical settings. The evolving field of molecular imaging requires development of a novel class of MR-detectable agents that can provide image contrast to target specific disease processes [55]. However, conventional DCE and DWI are still of topical interest, and they provide the most relevant and available MR biomarkers in today clinical practice and research [56–63].

**Fig. 8:**
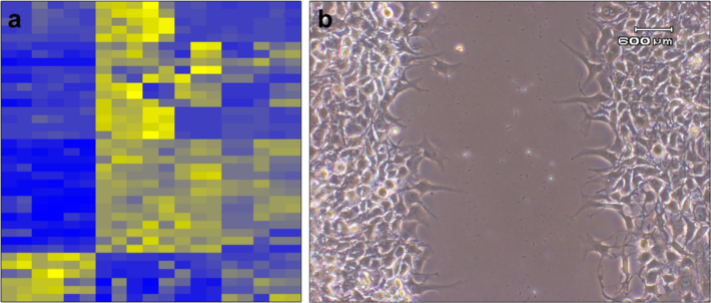
Tissue-based imaging. miRNA array analysis differentiates STS subtypes according to biological features and clinical response (**a**); wound healing assay allows to investigate cell-cell, cell-matrix interaction during cell migration to close the “wound” (**b**)

Tissue-based imaging strategies with and without prior fluorescent and bioluminescent labelling are currently employed in basic research providing the simultaneous discovery of phenotype-related key signatures. Competitive genomic hybridization array, based on competitive fluorescence, recognizes specific chromosome abnormalities associated with gain or loss of specific regions harbouring cancer progression-associated genes [64] and the integration with mRNA and miRNA array profiling (Fig. 8a) response [65] and the use of a reporter gene system may determine activity of a selected gene by visualizing post-transcriptional and post-translational events within downstream signalling pathways [66].

The impact of genetic changes on signalling pathway deregulation may be recognized by molecular imaging based on mass spectrometry. Mass spectrometry imaging (MALDI) technique uses matrix assisted laser desorption ionization where the sample, often a thin tissue section or a cell lysate, is moved in two dimensions while the mass spectrum is recorded. MALDI may identify regions of the proteome, as well as unmodified or post-translationally modified proteins or peptides, that today are a new area of interest for new biomarkers [16, 67, 68]. As complement of in vitro and ex vivo molecular biology assays, molecular imaging is also currently used in research animals for development of future clinical strategies providing a in vivo quantitative representation of the biological processes. By using intravital microscope we demonstrated that the capacity of fluorescently labelled LMS cells to invade lymphatic vessels in living mice [69] is increased by ectopic expression of proteoglycan NG2 involved in tumour cell-environment interaction, found also overexpressed in advanced/metastatic STS patients [70]. Deregulation of adhesion and invasion processes (Fig. 8b), degradation of extracellular matrix and the capability of tumour cells of migrating across endothelium is an essential pre-requisite for metastatic spread of tumour cells and represent one of the multiple hallmarks in tumour progression [71]. In high-grade STS characterized by tissue heterogeneity, cellular and molecular imaging may contribute to improve patient survival through in vivo detection of prognostic metabolic indicators and efficacy of treatment.

## Conclusions

Translation from laboratory characterization to clinical application passes through molecular imaging in patients. Measurement of biological endpoints and visualization of functional and metabolic changes to malignant transformation and progression provide early detection of disease and monitoring of therapy efficacy. Thus, the integration of cell and molecular biology, pathology, bioinformatics, molecular imaging, new radiological imaging techniques and clinical features may identify new indicators of disease or therapeutic effects through the quantitative representation of biological processes. In particular, this system-biology analysis may categorize subgroups of distinct endpoints involved in metastatic progression and drug-resistance. Up to now despite the advances in identifying gene abnormalities and deregulated pathways few specific endpoints represent direct targets in sarcoma treatment and chemotherapy remains the first choice treatment of advanced/metastatic sarcoma.
